# Sex Differences in Patient‐Reported Outcomes Among People Living With HIV Switching to an Oral Dual Therapy: Results From the PROBI Study

**DOI:** 10.1155/arat/1850783

**Published:** 2025-10-02

**Authors:** Tristan Alain, Fabienne Marcellin, Pascal Bessonneau, Laurent Hocqueloux, Holger Muehlan, Clotilde Allavena, David Zucman, Ester Villalonga-Olives, Olivier Chassany, Martin Duracinsky

**Affiliations:** ^1^ Patient-Reported Outcomes Research (PROQOL), Health Economics Clinical Trial Unit (URC-ECO), Hotel-Dieu Hospital, AP-HP, Paris, France, aphp.fr; ^2^ Economic and Social Sciences of Health & Medical Information Processing (SESSTIM), Aix Marseille University, Inserm, IRD, ISSPAM, Marseille, France, univ-amu.fr; ^3^ Infectious Disease Department, Orléans University Hospital, Orléans, France; ^4^ Health & Medical University Erfurt, Erfurt, Germany; ^5^ INSERM EA 1413, Infectious Diseases Department, Nantes University Hospital, Nantes, France, chu-nantes.fr; ^6^ Department of Internal Medicine, Foch Hospital, Suresnes, France; ^7^ Department of Practice, Sciences and Health Outcomes Research, University of Maryland, Baltimore, Maryland, USA, umaryland.edu; ^8^ ECEVE, UMR-S 1123, Paris Cité University, Inserm, Paris, France, ciup.fr; ^9^ Internal Medicine Department, Bicêtre Hospital, AP-HP, Le Kremlin-Bicêtre, France, aphp.fr

## Abstract

**Introduction:**

Socioeconomic, behavioral, and psychosocial factors—beyond just biological and hormonal factors—drive sex differences in HIV outcomes. The PROBI study evaluated treatment acceptability, perceived toxicity, and health‐related quality of life (HRQL) among people living with HIV (PLWH) switching from multiple antiretroviral therapy to oral dual therapy. Higher treatment discontinuation rates were observed among women, prompting this analysis of sex‐based HRQL differences.

**Methods:**

HRQL, treatment acceptability, and symptom burden data were collected at treatment switch (D0) and at 1 and 6 months afterward (M1 and M6). Higher scores indicated greater symptom burden (HIV‐SI), better HRQL (PROQOL‐HIV), or better treatment acceptability. Sex differences were analyzed using appropriate statistical tests, with overtime changes assessed via mixed‐effects linear regression models.

**Results:**

The study included 260 PLWH (35% women, *n* = 92), with a mean age of 51 ± 12 years. Compared to men, women were more frequently born in sub‐Saharan Africa (46% vs. 12%), had lower educational attainment (20% vs. 41% with university degrees), and higher rates of obesity (29% vs. 9% with BMI ≥ 30). While no virologic failures occurred, treatment discontinuation was higher among women (15% vs. 5%). Over time, all participants showed improvements in symptom burden (−2 points), treatment‐related HRQL (+5 points), mental/cognitive HRQL (+4 points), and treatment acceptability (+6 points). However, women consistently demonstrated worse cognitive and mental HRQL scores compared to men (mean difference: −7 points).

**Conclusion:**

While dual therapy improved treatment acceptability and HRQL for both sexes, women maintained lower mental and cognitive HRQL scores. Enhanced patient‐provider communication may help identify HRQL changes, especially among women who face higher treatment discontinuation risk.

**Trial Registration:**

ClinicalTrials.gov identifier: NCT04788784

## 1. Introduction

Differences between men and women have been identified through the successive phases of HIV infection, from viral transmission to pathogenesis and treatment [1]. Compared with men, women face a higher risk of heterosexual transmission of HIV, especially in sub‐Saharan Africa [2]. In France, in 2023, 32% of newly diagnosed HIV infections concerned women, and 38% of newly diagnosed people were born in sub‐Saharan Africa [3]. Women also exhibit stronger immune activation in the early phases of the disease, with both lower plasma HIV viral loads and higher CD4 T‐cell counts [4, 5]. In high‐resource countries with extended access to highly active antiretroviral therapy (ART), their disease progression is slower than that of men [6]. In addition, even if virological efficacy of ART appears to be similar in men and women living with HIV [7], sex differences in pharmacokinetics and pharmacodynamics of some antiretroviral drugs have been reported [8]. Furthermore, women living with HIV are exposed to a higher risk of certain clinical events, such as weight gain following ART initiation [9–12], or bone fracture and osteoporosis, especially in the postmenopausal period [13–15]. They also face specific mental health issues [16] and report lower adherence to ART than men [17, 18]. All these sex differences are driven not only by biological and hormonal factors but also by socioeconomic, behavioral, and psychosocial ones [19].

Patient‐reported outcomes (PROs)—i.e., self‐reported measures of a patient’s health perception—have become key criteria guiding clinicians in choosing the best treatment options for people living with HIV (PLWH) [20]. Documenting sex differences in PLWH’s experience with ART is thus a key issue. There is a need for more data on PROs in women living with HIV, who remain underrepresented in clinical trials [21, 22]. As they reduce the number of medications required to manage HIV while maintaining durable virological efficacy, two‐drug regimens are now considered a viable alternative to three‐drug ones and are part of the standard of care [23, 24].

The Patient‐Reported Outcomes HIV BItherapy (PROBI) mixed‐method study [25] aimed to evaluate treatment acceptability, perceived toxicity, patients’ preferences, and health‐related quality of life (HRQL) from the patients’ perspective among PLWH switching from a standard three‐ or four‐drug ART regimen to the DTG/3TC‐combined therapy Dovato (one tablet per day with no food restriction), a therapeutic option recommended in maintenance therapy in ART‐experienced PLWH and in ART‐naive PLWH [24, 26]. Data from self‐administered questionnaires filled in at the time of the treatment switch, then one and 6 months later showed a global improvement in PROs which was shown after treatment switch [27].

A higher rate of treatment discontinuation was observed

among women. The present study aims to explore sex differences in treatment acceptability, perceived toxicity, preferences, and HRQL of participants who switched from a standard three‐ or four‐drug ART regimen to the dolutegravir‐ and lamivudine (DTG/3TC)‐combined therapy Dovato.

## 2. Materials and Methods

### 2.1. Study Design

The multicenter, longitudinal, noncomparative, observational, real‐life cohort study PROBI was conducted from April 2021 to May 2023 among adult PLWH (age ≥ 18 years) who were receiving a three‐ or four‐antiretroviral drug regimen, and who had an undetectable plasma HIV viral load (HIV‐RNA < 50 copies [cp]/mL) for at least three months. Other inclusion criteria were the prescription of Dovato by a clinician in routine care and the cognitive and linguistic ability to complete several self‐administered questionnaires. The main exclusion criteria comprised being a pregnant or breastfeeding woman, known hypersensitivity or resistance to DTG or 3TC, had active stage C HIV disease, according to the Centers for Disease Control and Prevention (CDC) classification, coinfection with hepatitis B virus (HBV), and having received parenteral antibiotic, antifungal therapy, radiation therapy, or cytotoxic chemotherapeutic agents during 30 days prior to inclusion. The PROBI study included a 6‐month follow‐up of PLWH in 25 medical centers across France (10 located in the Paris area and 15 in other French regions).

### 2.2. Data Collection

Sociodemographic, clinical (including blood test results), and therapeutic data were collected from medical records using electronic case report forms. PROs and data concerning PLWH’s behaviors were collected using electronic or paper self‐administered questionnaires. Data collection was scheduled at baseline (Day 0, D0), which corresponded to the day of the switch to Dovato, then at Month 1 (M1) and Month 6 (M6) of follow‐up. Further methodological details and primary findings are described elsewhere [27].

### 2.3. Sociodemographics

Collected sociodemographic information included sex at birth (men, women, transmen, and transwomen), age at inclusion, professional status (active and not active), educational level (none or primary education, secondary education, and university education), marital status (married or living with a partner, single, divorced, separated, or widowed), personal situation (living with other people and living alone), region of birth (Europe, sub‐Saharan Africa, and other regions), perceived financial situation (sufficient sufficient but needs to be careful, difficult, insufficient without borrowing money, and does not want to reply), body mass index (< 20.0, 20.0–24.9, 25.0–29.9, and ≥ 30.0), anti‐HCV antibody positivity (yes and no), current and/or past depression (yes and no), comorbidities (cardiovascular comorbidities including hypertension, dyslipidemia, diabetes, cirrhosis or severe liver failure, renal failure with clearance less than 60, other current, or past comorbidity), number of comorbidities, antidepressant treatment (yes and no), anxiolytic treatment (yes and no), HIV duration (years), CDC classification stage C (yes and no), HIV transmission group (heterosexual, homo‐ or bisexual, and others), CD4 nadir value (cells/mm^3^), total duration of HIV treatment (years), number of antiretroviral lines before switching, last type of antiretroviral drug regimen before the switch (INI + 2 NRTI, INI + 2 NRTI [TRIUMEQ], NNRTI + 2 NRTI, PI + 1 or 2 NRTI, and other treatment), frequency of treatment intake (every day and four or five days a week), and reason for switch (simplification, prevention of long‐term toxicity, decrease of side effects, and others).

### 2.4. HRQL

HRQL was assessed using the PROQOL‐HIV questionnaire [28], a validated HIV‐specific instrument with a 2‐week recall period, which is sensitive to the individual’s sociocultural context, disease stage, and antiretroviral treatment. The PROQOL‐HIV explores the following four global dimensions of HRQL: physical HRQL and symptoms (11 items), relationships with others (social, sexual, and self‐image) (7 items), mental and cognitive HRQL (10 items), and treatment impact (10 items). Associated scores range from 0 to 100, with higher values denoting better HRQL.

### 2.5. Treatment Acceptability

Treatment acceptability was assessed using the treatment acceptability scale (4 items, one dimension) that measures satisfaction, ease of use, desire to continue treatment with this new regimen, and recommendation to family and friends. Associated scores range from 0 to 100, with higher values denoting greater acceptability.

### 2.6. Perceived Toxicity and Preferences (PTPs)

PTP for oral dual therapy were assessed using the PTP scale (6 items, two dimensions: (i) preference for treatment and (ii) lifestyle convenience and perceived efficacy) [25]. Associated scores range from 0 to 100, with higher values denoting lower toxicity and higher preference.

### 2.7. Self‐Reported Symptoms

Self‐reported symptoms were assessed using 24 items (one dimension) derived from the HIV Symptom Index [29]. Each item in the symptom questionnaire was coded from 0 to 4 (0: *no symptom*, 1: *symptom without bother*, 2: *bothers a little*, 3: *bothers*, and 4: *bothers a lot*). A global score (standardized from 0 to 100) was calculated for all item scores, with higher values denoting a higher burden of symptoms (accounting for both the presence of each symptom and the associated discomfort).

### 2.8. Statistical Analyses

Analyses were restricted to participants in the PROBI study who started the Dovato therapy and filled in the self‐administered questionnaires. Sociodemographic and clinical characteristics of PLWH in the study population were described at D0 using numbers and percentages. Sex differences for continuous variables were tested using the Wilcoxon rank sum test after checking their distribution and rejecting the normality assumption. Sex differences for categorical variables were tested using Pearson’s chi‐square test or Fisher’s exact test [30] (when the counts for certain variable categories were less than 5). The number and percentage of discontinuations of the Dovato regimen, as well as reasons for discontinuation, were analyzed for the overall population and compared between men and women using Fisher’s exact test. The distribution of PRO scores was described at each visit using means and standard deviations (SDs). A potential sex effect on longitudinal HRQL, treatment acceptability, PTP, and self‐reported symptoms measures was tested using mixed‐effects linear regression models, with individual as the random effect. Mixed‐effects linear regression models [31, 32] are suitable for repeated measures as they account for correlations within individual observations. The distributions of residuals and random effects were checked to ensure that the assumptions required for applying such models were satisfied. Blood test results were described at D0 and M6, then compared between men and women using Wilcoxon rank sum test and Fisher’s exact test. The significance threshold was fixed at alpha = 0.05 in all analyses. Analyses were performed using R Version 4.3.3 [33].

### 2.9. Minimizing Bias Associated With Self‐Reports

Answers to self‐administered questionnaires may be subject to social desirability bias, which occurs when respondents answer questions in a way they think is more socially acceptable or favorable. The PRO scales used in the PROBI study were specifically designed and validated to minimize such bias (nonjudgmental wording, preambles that emphasize the value of honest answers, cultural adaptation, and pilot surveys to test the questionnaires) [28, 29, 34]. In addition, participants were informed that their answers will not be communicated to the medical staff and used only for research purposes.

### 2.10. Ethical Considerations

Ethical approval was obtained from an Independent Ethics Committee (Comité de Protection des Personnes Sud‐Méditerranée, France, study approval 2020‐A03514‐35). All participants provided oral consent to participate, confirmed in the questionnaire, and registered in patients’ files.

## 3. Results

### 3.1. Main Characteristics of the Study Population

Among the 266 PLWH initially enrolled in the PROBI study, 6 participants excluded were linked to exclusion criteria (1 had HBV coinfection, 2 did not start treatment with Dovato, 2 lost of follow‐up, and 1 could not fill in the questionnaires). The study population thus comprised a total of 260 PLWH. Among them, 168 were men (64.6%) and 92 were women (35.4%), including three transwomen.

At D0, women and men were similar in age, personal situation, and professional status (Table 1). Compared with men, women were more often born in sub‐Saharan Africa (45.7% vs. 11.9%, *p* < 0.001 for the overall comparison of birth regions), had a lower educational level (19.8% of women with university degree vs. 40.5% of men, *p* < 0.001), were more often divorced/separated or widowed (28.6% vs. 6.5%, *p* < 0.001), and were more often overweight (BMI ≥ 30) (29.2% vs. 8.9%, *p* < 0.001). Women also tended to report a worse financial situation than men (20.9% of women perceived themselves in difficult financial situation vs. 13.0% of men). However, this last difference was not statistically significant (*p* = 0.058). No significant sex difference was detected at D0 regarding the percentage of PLWH with a history of cured or active hepatitis C, depression, and other comorbidities, as well as intake of antidepressant or anxiolytics.

**Table 1:** Sociodemographic and clinical characteristics of people living with HIV by sex in the study population before switching to Dovato (*n* = 260, PROBI, Day 0).

Characteristics	Total number *N* = 260	Men; *n* (%) *N* = 168	Women; *n* (%) *N* = 92	*p* value^‡^
Age (years)^†^	260	*51 (12)*	*50 (11)*	0.272
Professional status	259			0.367
Active		111 (66.1%)	55 (60.4%)	
Not active		57 (33.9%)	36 (39.6%)	
Educational level	259			**< 0.001**
No or primary education		10 (6.0%)	27 (29.7%)	
Secondary education		90 (53.6%)	46 (50.5%)	
University education		68 (40.5%)	18 (19.8%)	
Marital status	259			**< 0.001**
Married/living with a partner		86 (51.2%)	38 (41.8%)	
Single		71 (42.3%)	27 (29.7%)	
Divorced/separated or widowed		11 (6.5%)	26 (28.6%)	
Personal situation	259			0.163
Lives with other people		92 (54.8%)	58 (63.7%)	
Lives alone		76 (45.2%)	33 (36.3%)	
Birth region	260			**< 0.001**
Europe		128 (76.2%)	43 (46.7%)	
Sub‐Saharan Africa		20 (11.9%)	42 (45.7%)	
Other regions		20 (11.9%)	7 (7.6%)	
Perceived financial situation	253			0.058
Sufficient		99 (61.1%)	42 (46.2%)	
Sufficient but needs to be careful		37 (22.8%)	22 (24.2%)	
Difficult		21 (13.0%)	19 (20.9%)	
Insufficient without borrowing money		4 (2.5%)	5 (5.5%)	
Does not want to reply		1 (0.6%)	3 (3.3%)	
Body mass index	257			**< 0.001**
< 20.0		17 (10.1%)	9 (10.1%)	
20.0–24.9		86 (51.2%)	28 (31.5%)	
25.0–29.9		50 (29.8%)	26 (29.2%)	
≥ 30.0		15 (8.9%)	26 (29.2%)	
Anti‐HCV antibody positive	260	18 (10.7%)	5 (5.4%)	0.152
Current and/or past depression	260	35 (20.8%)	21 (22.8%)	0.709
Comorbidities				
Cardiovascular comorbidities, including hypertension	260	44 (26.2%)	19 (20.7%)	0.319
Dyslipidemia	260	52 (31.0%)	22 (23.9%)	0.229
Diabetes	260	15 (8.9%)	7 (7.6%)	0.715
Cirrhosis or severe liver failure	260	2 (1.2%)	0 (0.0%)	0.541
Renal failure, clearance less than 60	260	11 (6.5%)	4 (4.3%)	0.467
Other current or past comorbidity	260	36 (21.4%)	28 (30.4%)	0.107
Number of comorbidities^†^	260	*1 (1)*	*1 (1)*	0.407
Antidepressant treatment	260	13 (7.7%)	10 (10.9%)	0.395
Anxiolytic treatment	260	13 (7.7%)	7 (7.6%)	0.970
HIV duration (years)^†^	260	*16 (10)*	*17 (8)*	0.248
CDC classification Stage C	260	34 (20.2%)	16 (17.4%)	0.578
HIV transmission group	260			**< 0.001**
Heterosexual		44 (26.2%)	82 (89.1%)	
Homo‐bisexual		113 (67.3%)	4 (4.3%)	
Others^§^		11 (6.5%)	6 (6.5%)	
CD4 nadir value (cells/mm^3^)^†^	258	*280 (213)*	*284 (165)*	0.500
Total duration of HIV treatment (years)^†^	260	*13 (8)*	*15 (7)*	0.058
Number of antiretroviral lines before switching^†^	259	*4 (3)*	*6 (4)*	**< 0.001**
Last type of antiretroviral drug regimen before the switch	260			0.065
INI + 2 NRTI		67 (39.9%)	27 (29.3%)	
INI + 2 NRTI (TRIUMEQ®)		58 (34.5%)	36 (39.1%)	
NNRTI + 2 NRTI		36 (21.4%)	19 (20.7%)	
PI + 1 or 2 NRTI		4 (2.4%)	9 (9.8%)	
Other treatment		3 (1.8%)	1 (1.1%)	
Frequency of treatment intake	259			0.717
Every day		162 (96.4%)	89 (97.8%)	
4 or 5 days/week		6 (3.6%)	2 (2.2%)	
Reason for switch (specified by the clinician)	260	168 (100.0%)	92 (100.0%)	> 0.999
Simplification	260	146 (86.9%)	80 (87.0%)	0.991
Prevention of long‐term toxicity	260	29 (17.3%)	21 (22.8%)	0.276
Decrease of side effects	260	37 (22.0%)	11 (12.0%)	**0.045**
Others	260	5 (3.0%)	2 (2.2%)	> 0.999

*Note:* INI = integrase inhibitor. Significant *p* values (> 0.05) are shown in bold.

Abbreviations: NNRTI = non‐nucleoside reverse transcriptase inhibitor, NRTI = nucleoside reverse transcriptase inhibitor, and PI = protease inhibitor.

^†^Mean (standard deviation) in italic.

^‡^Wilcoxon rank sum test; Pearson’s Chi‐squared test; Fisher’s exact test.

^§^Other HIV transmission groups: injection drug use *n* = 9, transfusion, or medical procedure *n* = 3, mother‐to‐child transmission *n* = 2, and unknown *n* = 3.

The HIV heterosexual transmission mode was more represented in women (89.1% vs. 26.2%) while the homo‐bisexual transmission mode was more represented in men (67.3% vs. 4.3%, *p* < 0.001 for the overall comparison of transmission groups). Globally, the mean ± SD time since HIV diagnosis at D0 was 16 ± 10 years, 19.2% of PLWH presented a CDC Stage C, the mean total duration of HIV treatment was 14 ± 8 years, and the mean CD4 nadir value was 282 ± 197 cells/mm^3^, with no sex differences.

Women had received more antiretroviral regimens before switching to Dovato with a mean ± SD of 6 ± 4 vs. 4 ± 3 for men (*p* < 0.001). The predominant reasons for switching to Dovato reported by clinicians were treatment simplification (86.9%) and prevention of long‐term toxicity (19.2%). Switching for adverse events of the previous treatment concerned more men than women (22% vs. 12%, *p* = 0.045) (Table 1).

Before the switch to Dovato, 99% of PLWH had undetectable viral load, with no difference between men and women. Detailed blood test results can be consulted in Supporting Data Table 1.

### 3.2. Treatment Discontinuations

Twenty participants stopped treatment during the study, with women being more likely to discontinue than men (13%, *n* = 12 vs. 5%, *n* = 8; *p* = 0.004) (Table 2). Reasons for discontinuation were most often linked to adverse events (16/20) with no difference between men and women concerning the type of adverse event (neuropsychiatric disorders (*n* = 5), digestive disorders (*n* = 4), and osteoarticular disorders (*n* = 3).

**Table 2:** Description of DTG/3TC (dovato) treatment discontinuations among people living with HIV participating in the PROBI study (*n* = 260).

Treatment discontinuation characteristics	Men *N* = 8	Women *N* = 12	*p* value^‡^
Discontinuation visit			0.650
Month 1 visit	5	5	
Month 6 visit	3	7	
Reasons for discontinuation			
Weight gain	1	1	> 0.999
Neuropsychiatric disorders	2	3	> 0.999
Digestive disorders	1	3	0.619
Osteoarticular disorders	2	1	0.537
Adherence	1	0	0.400
Patient request^†^	1	2	> 0.999
Last type of antiretroviral drug regimen before the switch			> 0.999
INI + 2 INTI	5	6	
INI + 2 INTI (TRIUMEQ)	0	1	
INNTI + 2 INTI	2	4	
IP + 2 INTI	1	1	

*Note:* INI = integrase inhibitor.

Abbreviations: NNRTI = non‐nucleoside reverse transcriptase inhibitor, NRTI = nucleoside reverse transcriptase inhibitor, and PI = protease inhibitor.

^†^Patient request: discontinuation based on request from the patient.

^‡^Fisher’s exact test.

### 3.3. Longitudinal Outcome Analysis

The distributions of PRO scores by sex at each visit are presented in Table 3.

**Table 3:** Distribution of outcome scores for people living with HIV by sex during follow‐up in the PROBI study (*n* = 260).

Scales	Visit D0 *N* = 253			Visit M1 *N* = 239			Visit M6 *N* = 229		
	Missing	Men mean (SD) *N* = 162	Women mean (SD) *N* = 91	Missing	Men mean (SD) *N* = 156	Women mean (SD) *N* = 83	Missing	Men mean (SD) *N* = 148	Women mean (SD) *N* = 81
PROQOL HIV dimensions for assessment of health‐related quality of life^†^									
Physical HRQL and symptoms	0	82 (17)	81 (18)	0	83 (16)	83 (15)	1	84 (15)	81 (17)
Relationships with others (social, sexual, and self‐image)	0	84 (17)	81 (23)	1	85 (17)	84 (19)	1	86 (17)	83 (20)
Mental and cognitive HRQL	0	72 (21)	64 (27)	1	74 (21)	68 (24)	0	76 (20)	70 (21)
Treatment impact	0	84 (15)	82 (16)	0	88 (13)	87 (12)	0	89 (11)	87 (11)
Acceptability of treatment^†^									
Acceptability dimension	1	75 (23)	74 (21)	1	81 (21)	78 (20)	1	81 (21)	81 (17)
Perceived toxicity and preference for treatment (PTP)^†^									
Lifestyle convenience and perceived efficacy dimension	1	86 (15)	87 (15)	2	84 (14)	83 (14)	1	84 (18)	87 (12)
Preference for dual therapy dimension	1	73 (22)	71 (25)	1	74 (23)	74 (23)	1	73 (23)	72 (23)
Self‐reported symptoms (HIV Symptom Index)^‡^									
Global score	2	14 (13)	15 (15)	2	12 (12)	11 (11)	2	13 (12)	11 (14)

*Note:* D0 = Day 0; M1 = Month 1; M6 = Month 6.

Abbreviations: HRQL = health‐related quality of life, PROs = patient‐reported outcomes, and SD = standard deviation.

^‡^Scores between 0 (lowest symptom burden) and 100 (highest symptom burden).

^†^Scores between 0 (worse HRQL) and 100 (best HRQL).

Concerning HRQL, mean PROQOL‐HIV scores during follow‐up were above 80 (on a 0 to 100 scale) for all dimensions except mental and cognitive HRQL. For the latter, mean (SD) scores were 72 (21) for men and 64 (27) for women at D0, then reached 76 (20) for men and 70 (21) for women at M6. Without sex differences, mean scores during follow‐up varied globally, from minimum to maximum, between 74 (21) and 81 (21) for acceptability of treatment, 71 (25) and 74 (23) for preference for dual therapy, 83 (14) and 87 (15) for lifestyle convenience and perceived efficacy, and between 11 (11) and 15 (15) for self‐reported symptoms (HIV Symptom Index global score).

**Figure 1:**
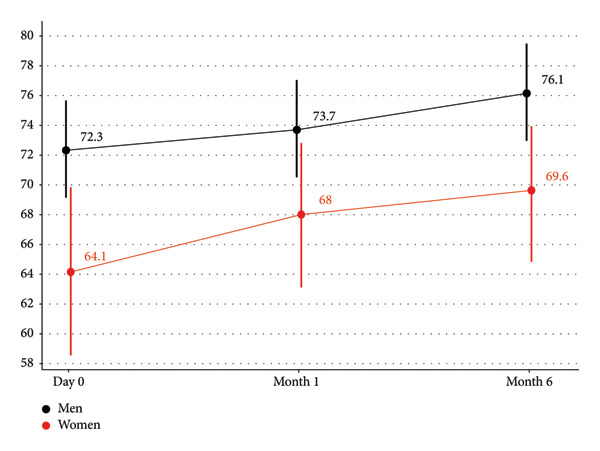
Mean (standard deviation) scores changes from D0 to M6 of people living with HIV for the mental and cognitive HRQL dimension from PROQOL‐HIV scale by sex, PROBI study, *n* = 260.

Overtime, a significant increase was detected in mental and cognitive HRQL +4.2 (+2.1; +6.4), treatment impact‐related HRQL +5.1 (+3.5; +6.7), and treatment acceptability +6.0 (+2.9; +9.1), as shown by the estimated coefficients associated with the “Visit” variable in the mixed‐effects linear regression models (Table 4). By contrast, a significant decrease was detected for the global HIV Symptom Index score −2.1 (−3.4; −0.7). Significant sex differences were observed only for the cognitive and mental HRQL dimension scores for women compared with men (mean change: −7.0 [−12.0; −1.9]) (Figure 1). The assumptions required for the use of mixed‐effects linear regression were satisfied for all models. Supporting Figure 1 presents, as an example, the diagnostic distribution plots for the model with the mental/cognitive HRQL dimension score as the outcome.

**Table 4:** Longitudinal analyses of outcome scores during people living with HIV follow‐up in the PROBI study (*n* = 260, mixed‐effects linear regression models).

Models by PRO score^†^	Variables	Beta	95% CI	*p* value
*HRQL (PROQOL HIV scale)*				
Physical HRQL and symptoms dimension	Score at visit (ref M0)			0.3
	M1	1.3	−0.34, 2.9	
	M6	0.92	−0.72, 2.6	
	Sex (ref men)			0.5
	Women	−1.3	−5.1, 2.5	
Relationships with others (social, sexual, and self‐image) dimension	Score at visit (ref M0)			0.11
	M1	1.6	−0.36, 3.6	
	M6	2.0	−0.03, 4.0	
	Sex (ref men)			0.2
	Women	−3.1	−7.3, 1.2	
Mental and cognitive dimension	Score at visit (ref M0)			**< 0.001**
	M1	2.1	0.03, 4.2	
	M6	4.2	2.1, 6.4	
	Sex (ref men)			**0.008**
	Women	−7.0	−12, −1.9	
Treatment impact dimension	Score at visit (ref M0)			**< 0.001**
	M1	4.9	3.4, 6.5	
	M6	5.1	3.5, 6.7	
	Sex (ref men)			0.3
	Women	−1.7	−4.6, 1.3	

*Acceptability (acceptability scale)*				
Acceptability dimension	Score at visit (ref M0)			**< 0.001**
	M1	5.2	2.2, 8.3	
	M6	6.0	2.9, 9.1	
	Sex (ref men)			0.5
	Women	−1.4	−5.4, 2.7	

*Perceived toxicity and preference for oral dual therapy (PTP scale)*				
Preference for treatment and lifestyle convenience dimension	Score at visit (ref M0)			0.11
	M1	−2.2	−4.3, 0.03	
	M6	−1.8	−4.0, 0.38	
	Sex (ref men)			0.6
	Women	0.71	−2.3, 3.7	
Perceived efficacy dimension	Score at visit (ref M0)			0.4
	M1	1.8	−1.3, 5.0	
	M6	−0.20	−3.4, 3.0	
	Sex (ref men)			0.6
	Women	−1.3	−6.0, 3.4	

*Self-reported symptoms (HIV Symptom Index)*				
Global HIV Symptom Index score	Score at visit (ref M0)			**< 0.001**
	M1	−3.3	−4.6, −2.0	
	M6	−2.1	−3.4, −0.71	
	Sex (ref men)			0.8
	Women	0.33	−2.6, 3.3	

*Note:* Significant *p* values (> 0.05) are shown in bold.

Abbreviations: CI = confidence interval, HRQL = health‐related quality of life, and PROs = patient‐reported outcomes.

^†^For each score associated with a PROs dimension, the table presents beta coefficients and 95% CI for the multivariable model adjusted for time (visit) and sex.

## 4. Discussion

Findings from this real‐life, multicenter study conducted among 260 adult PLWH (=> 18 years old) highlight a global improvement of PROs, including health‐related quality of life, acceptability, treatment preference, and self‐reported symptoms in both men and women, during a 6‐month follow‐up after switching from a standard three‐ or four‐antiretroviral drug regimen to the DTG/3TC dual therapy Dovato. A statistically significant improvement was observed at Month six postswitch in mental and cognitive HRQL, treatment impact‐related HRQL, treatment acceptability, and self‐reported symptoms in both men and women. Despite the improvement, mental and cognitive HRQL scores remained lower in women compared with men throughout follow‐up. Women living with HIV have been found to present a higher burden of mental health issues, such as anxiety or depression, than their male counterparts [16]. Even if the percentage of PROBI participants receiving antidepressant or anxiolytic treatment at D0 was relatively low, with no difference between men and women, the presence of untreated or undiagnosed anxiety or depressive symptoms cannot be discarded, with potentially more symptoms among women, which may contribute to the sex difference observed in mental and cognitive HRQL.

It is interesting to note that men and women who participated in the PROBI study had very different sociodemographic profiles at D0, with a large proportion of women native from sub‐Saharan Africa, while most men were native from Europe, a lower educational level among women (29.7% of them reported primary education or no education at all), and a higher percentage of women having no partner, of whom most of them were divorced or widowed. Socioeconomic vulnerability and lack of support from a partner may thus partially explain women’s poorer perceived mental HRQL [35]. Cultural differences in individuals’ perceptions and representations of HRQL, including differences in the importance given to various aspects of quality of life, may also play a role [36]. Due to these cultural differences, our findings may not be generalizable to broader global contexts. Future research is needed to explore sex differences in PROs associated with dual ART regimens in other regions of the world, including sub‐Saharan Africa, where a significant proportion of female participants originated. The observed increase over time in scores for treatment acceptability and treatment‐related HRQL, for both men and women, confirms the positive experience of treatment simplification for all study participants [27].

Another key finding of the present study is the higher rate of Dovato discontinuations found in women during follow‐up. This finding warrants the need for a close monitoring of women’s experience with treatment in the early phases after switching, to identify potential difficulties they may face to maintain adherence to therapy and to provide adequate support. Of note, the overall percentage of treatment discontinuations was relatively low in the PROBI study compared to other similar cohorts, as previously shown in other cohorts of PLWH receiving this dual therapy [37, 38]. Interestingly, very few treatment discontinuations were due to weight gain, a well‐known adverse event with dolutegravir‐including regimen, for both men and women, which is specifically interesting given the high rate of overweight women in the study, at higher risk of weight gain with dolutegravir.

Several strengths and limitations should be acknowledged for the present study. The real‐life nature of the study, its longitudinal and multicentric design and the exploration of different outcomes represent the main strengths of the study. The restriction of participation to France may however limit the generalizability of findings. In addition, social desirability bias in PLWH’s self‐reports may have influenced assessment of outcomes. However, to ensure data validity, the PROBI study utilized PRO scales that were developed with strategies aimed at minimizing social desirability bias, which is inherent in self‐reported data.

Lastly, the noncomparative design of the study limits the ability to explore the underlying causes of changes in PROs over time. Further studies incorporating a control group maintained on standard treatment are warranted to more precisely distinguish the effects attributable to the treatment switch from those due to other potential contributing factors, such as time effects or participant expectations.

## 5. Conclusion

Our findings confirm the acceptability of the combined dual therapy Dovato for both men and women living with HIV who present an undetectable viral load under three‐ or four‐drug ART, as an option to both reduce the number of drugs taken on a daily basis and improve overall treatment experience and HRQL. Despite the improvement, mental and cognitive HRQL scores remained lower in women compared with men throughout follow‐up. Patient‐provider exchanges in the first month after treatment switch may help identify and solve potential difficulties with maintaining adherence, especially among women, for whom the risk of treatment discontinuation is higher. Evaluation of mental health could also help identify the difficulties in this population. The implementation of practical tools—such as electronic applications that allow patients to complete PROs at home and securely share the data with healthcare providers prior to medical appointments [39]—may contribute to enhancing communication between patients and providers.

**Nomenclature**
DTG: Dolutegravir
HRQL: Health‐related quality of life
INI: Integrase inhibitor
NNRTI: Non‐nucleoside reverse transcriptase inhibitor
NRTI: Nucleoside reverse transcriptase inhibitor
PI: Protease inhibitor
PLWH: People living with HIV
PROs: Patient‐reported outcomes
PROQOL‐HIV: Patient‐reported outcomes quality of life–HIV
3TC: Lamivudine

## Ethics Statement

Ethical approval was obtained from an Independent Ethics Committee (Comité de Protection des Personnes Sud‐Méditerranée, France, study approval 2020‐A03514‐35). All participants provided oral consent to participate, which was documented in their medical records and further confirmed by them in the self‐reported questionnaire.

## Disclosure

The funder ViiV Healthcare was involved in the manuscript approval.

## Conflicts of Interest

Laurent Hocqueloux reports nonfinancial support from Gilead, Merck Sharp and Dohme, and ViiV Healthcare; honoraria payments and travel support for advisory board participation from Gilead, Merck Sharp & Dohme, and ViiV Healthcare; and personal consultation fees from Gilead, Merck Sharp and Dohme, and ViiV Healthcare, all outside the submitted work.

Clotilde Allavena received travel grants or honoraria from ViiV Healthcare, Gilead, and MSD, all outside the submitted work.

Martin Duracinsky received travel grants or honoraria from ViiV Healthcare, Gilead, and MSD, all outside the submitted work.

Olivier Chassany received travel grants or honoraria from ViiV Healthcare, Gilead, and MSD, all outside the submitted work. The remaining authors declare no conflicts of interest.

## Author Contributions

Conceptualization: Laurent Hocqueloux, Clotilde Allavena, David Zucman, Olivier Chassany, and Martin Duracinsky.

Methodology: Pascal Bessonneau, Olivier Chassany, and Martin Duracinsky.

Validation: Tristan Alain and Martin Duracinsky.

Formal analysis: Tristan Alain and Pascal Bessonneau.

Investigation: Laurent Hocqueloux, Clotilde Allavena, David Zucman, and Martin Duracinsky.

Data curation: Tristan Alain.

Writing–original draft preparation: Tristan Alain and Fabienne Marcellin.

Writing–review and editing: Tristan Alain, Fabienne Marcellin, Pascal Bessonneau, Laurent Hocqueloux, Holger Muehlan, Clotilde Allavena, David Zucman, Ester Villalonga‐Olives, Olivier Chassany, and Martin Duracinsky.

Visualization: Tristan Alain and Pascal Bessonneau.

Supervision: Tristan Alain, Fabienne Marcellin, and Martin Duracinsky.

Funding acquisition: Martin Duracinsky.

Ethical approval: Martin Duracinsky.

## Funding

This work was supported by ViiV Healthcare (grant numbers 10.13039/100010877) and sponsored by Association Robert Debré pour la Recherche Médicale (ARDRM).

## Supporting Information

Additional supporting information can be found online in the Supporting Information section.

## Supporting information


**Supporting Information 1** Supporting data Table 1: Distribution of blood test results by sex during follow‐up in the PROBI study.

**Supporting Information 2** Supporting data Figure 1: Diagnostic plots for the mixed‐effects linear regression model with the mental and cognitive HRQL dimension score as the outcome, PROBI study, *n* = 260.

## Data Availability

Data are available on request from the authors. The data that support the findings of this study are available from the corresponding author upon reasonable request.
