# Supplementary material for: Sex Differences in Patient‐Reported Outcomes Among People Living With HIV Switching to an Oral Dual Therapy: Results From the PROBI Study
Source: AIDS Res Treat. 2025 Oct 2;2025:1850783. doi: 10.1155/arat/1850783 (PMC12510766; doi:10.1155/arat/1850783)
Supplement: Supplementary file 1 — Supporting Information 1 Supporting data Table 1: Distribution of blood test results by sex during follow‐up in the PROBI study. [file ARAT-2025-1850783-s001.docx]

**Supplementary data table 1: Distribution of blood tests results of people living with HIV by sex during follow-up in the PROBI study**

| **Blood tests** | **Visit D0** N=260 | | | | **Visit M6** N=250 | | | |
| --- | --- | --- | --- | --- | --- | --- | --- | --- |
|  | **N** | **Men**, mean (SD) N = 168 | **Women**, mean (SD) N = 92 | **p-value**^‡^ | **N** | **Men**, mean (SD) N = 162 | **Women**, mean (SD) N = 88 | **p-value**^‡^ |
| **HIV metrics** |  |  |  |  |  |  |  |  |
| Undetectable viral load (< 50 copies/mL) **^†^** | 260 | *167 (99.4 %)* | *91 (98.9 %)* | >0.999 | 241 | *157 (99.4 %)* | *82 (98.8 %)* | >0.999 |
| CD4 count (/mm3) | 259 | 708 (278) | 776 (317) | 0.145 | 220 | 720 (287) | 818 (315) | **0.023** |
| CD4 % | 257 | 35 (10) | 38 (9) | **0.038** | 221 | 34 (10) | 38 (9) | **0.017** |
| **Biology report** |  |  |  |  |  |  |  |  |
| blood creatinine (µmol/L) | 255 | 92.11 (15.56) | 72.92 (15.06) | **<0.001** | 231 | 93.01 (14.71) | 76.72 (15.20) | **<0.001** |
| creatinine clearance (ml/min/1.73m^2^) | 241 | 83.87 (16.50) | 87.98 (20.80) | 0.191 | 219 | 83.93 (15.29) | 84.42 (20.79) | 0.966 |
| creatinine clearance less than 50 mL/min/1.73m^2^ **^†^** | 241 | *3 (1.9 %)* | *2 (2.4 %)* | >0.999 | 219 | *3 (2.1 %)* | *3 (4.1 %)* | 0.403 |
| ALAT (U/I) | 251 | 30.37 (17.42) | 22.34 (10.87) | **<0.001** | 227 | 28.73 (14.66) | 23.48 (16.68) | **<0.001** |
| ASAT (U/I) | 250 | 29.16 (13.30) | 24.57 (10.74) | **0.001** | 224 | 28.78 (12.77) | 24.41 (7.34) | **0.033** |
| **Lipid profile** |  |  |  |  |  |  |  |  |
| Fasting blood glucose (mmol/L) | 223 | 5.68 (1.43) | 5.45 (1.04) | 0.093 | 173 | 5.76 (1.97) | 5.44 (1.40) | 0.210 |
| LDL (mmol/L) | 227 | 2.90 (1.04) | 3.20 (1.04) | **0.043** | 160 | 2.90 (0.96) | 3.33 (1.40) | 0.112 |
| HDL (mmol/L) | 231 | 1.33 (0.49) | 1.60 (0.57) | **<0.001** | 163 | 1.33 (0.32) | 1.61 (0.41) | **<0.001** |
| Triglycerides (mmol/L) | 232 | 1.49 (0.92) | 1.54 (1.16) | 0.511 | 170 | 1.43 (0.82) | 1.43 (1.05) | 0.409 |
| Total cholesterol (mmol/L) | 232 | 4.81 (1.14) | 5.32 (0.99) | **<0.001** | 170 | 4.91 (1.07) | 5.57 (1.62) | **0.012** |
| **^†^***Number of individuals (%)* | | | | | | | | |
| ^‡^Wilcoxon rank sum test; Fisher’s exact test |  |  |  |  |  |  |  |  |
| D0 = Day 0; M6 = Month 6; N = Number of patients; SD = Standard Deviation | | | | | | | | |
