# Supplementary material for: Sex Differences in Patient‐Reported Outcomes Among People Living With HIV Switching to an Oral Dual Therapy: Results From the PROBI Study
Source: AIDS Res Treat. 2025 Oct 2;2025:1850783. doi: 10.1155/arat/1850783 (PMC12510766; doi:10.1155/arat/1850783)
Supplement: Supplementary file 2 — Supporting Information 2 Supporting data Figure 1: Diagnostic plots for the mixed‐effects linear regression model with the mental and cognitive HRQL dimension score as the outcome, PROBI study, n = 260. [file ARAT-2025-1850783-s002.docx]

**
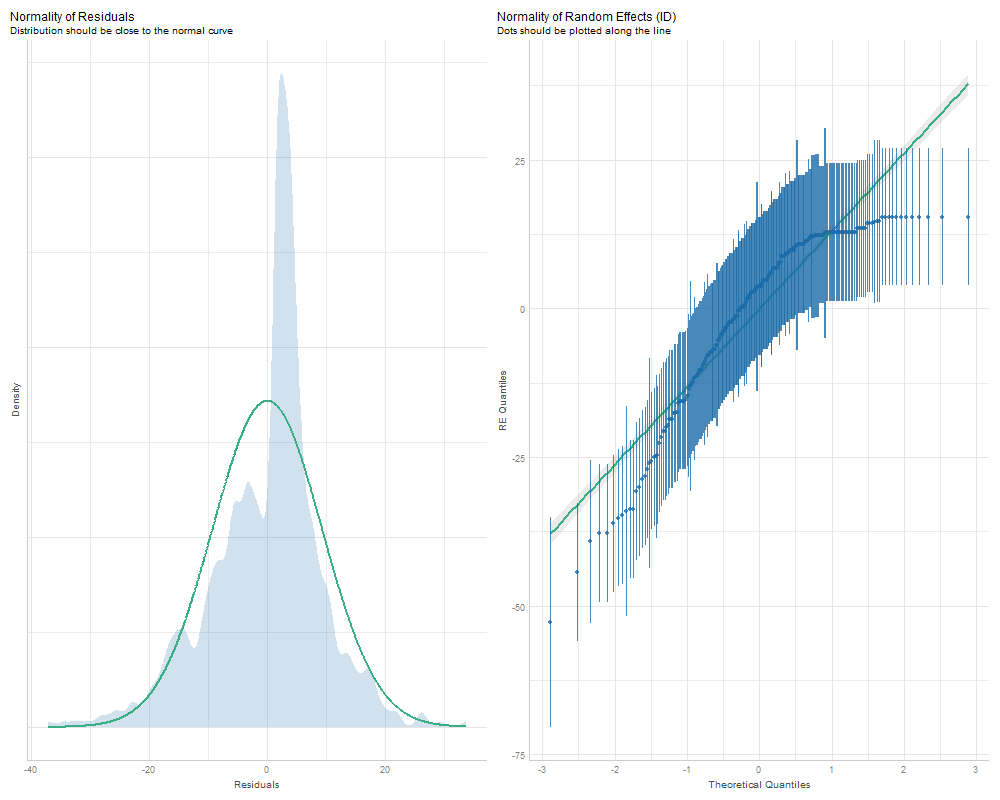
**

**Supplementary Figure 1: Diagnostic plots for the mixed-effects linear regression model with the mental and cognitive HRQL dimension score as the outcome, PROBI study, n=260**
